# Supplementary figures and images for: Comprehensive characterization of the bacterial community structure and metabolite composition of food waste fermentation products via microbiome and metabolome analyses
Source: PLoS One. 2022 Mar 15;17(3):e0264234. doi: 10.1371/journal.pone.0264234 (PMC9048815; doi:10.1371/journal.pone.0264234)

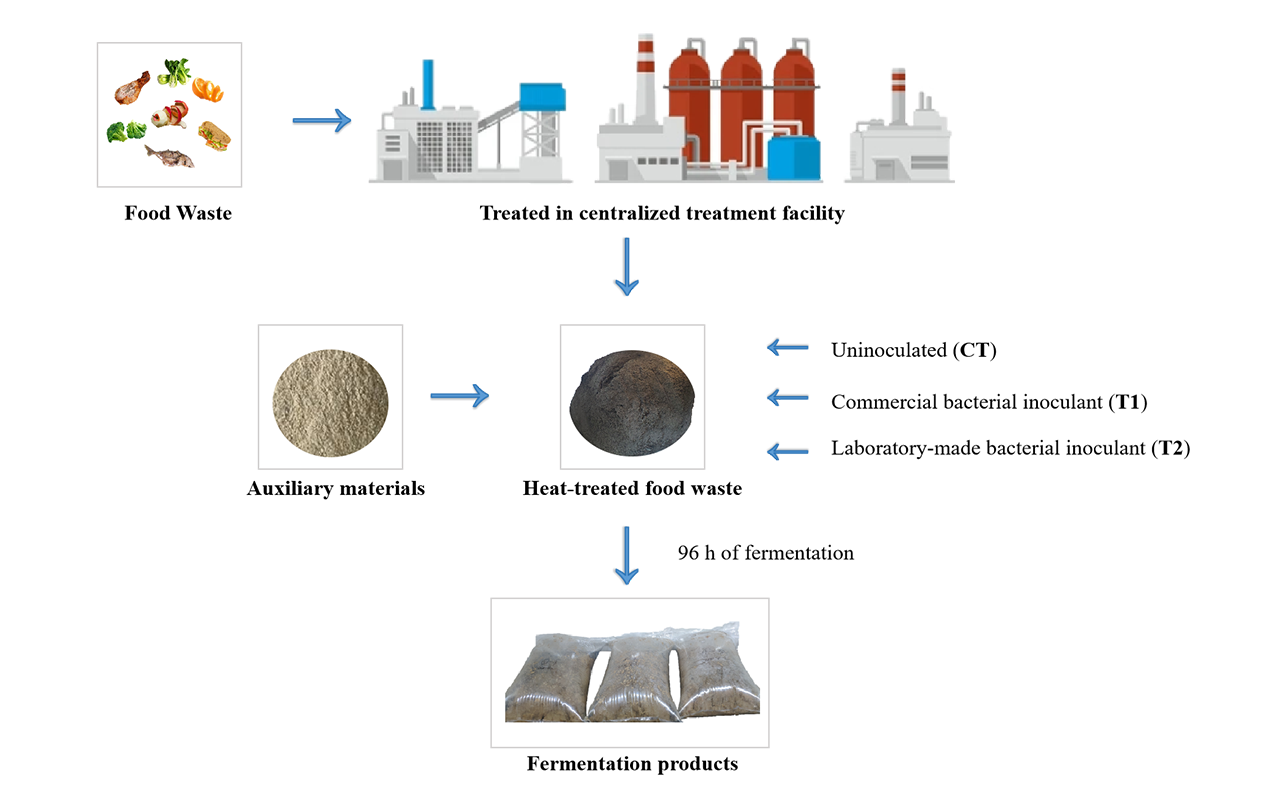

Supplement: S1 Fig — (TIF) [file pone.0264234.s001.tif]
