# Supplementary material for: Comprehensive characterization of the bacterial community structure and metabolite composition of food waste fermentation products via microbiome and metabolome analyses
Source: PLoS One. 2022 Mar 15;17(3):e0264234. doi: 10.1371/journal.pone.0264234 (PMC9048815; doi:10.1371/journal.pone.0264234)
Supplement: S1 Table — (DOCX) [file pone.0264234.s002.docx]

**S1 Table. Identification of significant key metabolites by LC-MS in fermented food waste sample.**

|  | **Relative concentration** | | | **Fold-changes** | | |
| --- | --- | --- | --- | --- | --- | --- |
| **Metabolite name** | **CT** | **T1** | **T2** | **Log_2_ (T1/CT)** | **Log_2_ (T2/CT)** | **Log_2_ (T1/T2)** |
| L-Valine | 0.012 | 0.016 | 0.018 | 0.423** | 0.593** | -0.170* |
| L-Tyrosine | 0.001 | 0.001 | 0.001 | 0.130** | 0.408** | -0.278** |
| L-Phenylalanine | 0.024 | 0.030 | 0.031 | 0.302** | 0.363** | -0.061 |
| L-Methionine | 0.003 | 0.004 | 0.004 | 0.457** | 0.673** | -0.215* |
| L-Lysine | 0.002 | 0.001 | 0.003 | -0.244 | 0.665** | -0.909** |
| L-Isoleucine | 0.011 | 0.016 | 0.017 | 0.516** | 0.598** | -0.082 |
| L-Glutamic acid | 0.002 | 0.001 | 0.002 | -0.554** | -0.554 | -0.612** |
| L-Aspartic acid | 0.001 | 0.001 | 0.003 | 0.349** | 1.401** | -1.052** |
| D-Proline | 0.007 | 0.010 | 0.008 | 0.585** | 0.202 | 0.383 |
| DL-Alanine | 0.001 | 0.002 | 0.002 | 0.899** | 0.778** | 0.120 |
| Beta-Leucine | 0.001 | 0.001 | 0.001 | 0.726* | 0.849** | -0.123 |
| Linoleic acid | 0.023 | 0.029 | 0.024 | 0.340** | 0.074 | 0.266* |
| 9-HODE | 0.001 | 0.001 | 0.005 | 0.379 | 2.348** | -1.969** |
| D-Lactic Acid | 0.061 | 0.089 | 0.084 | 0.549** | 0.471** | 0.078 |
| Xanthine | 0.000 | 0.003 | 0.003 | 3.115** | 3.330** | -0.216* |
| Guanine | 0.001 | 0.002 | 0.002 | 0.574 | 1.348** | 0.574** |
| Cytosine | 0.000 | 0.002 | 0.002 | 1.839** | 2.220** | -0.380** |
| Adenine | 0.002 | 0.003 | 0.006 | 0.630** | 1.540** | -0.911** |
| Trigonelline | 0.001 | 0.002 | 0.001 | 0.296** | -0.371** | 0.667** |
| Tartaric acid | 0.001 | 0.002 | 0.001 | 0.575** | 0.180 | 0.395** |
| Pyruvic acid | 0.001 | 0.002 | 0.002 | 1.105** | 1.352** | -0.247* |
| Phosphoric acid | 0.004 | 0.008 | 0.006 | 0.851** | 0.468 | 0.383* |
| Phenylacetaldehyde | 0.008 | 0.013 | 0.017 | 0.744** | 1.112** | -0.368** |
| Malonic acid | 0.001 | 0.002 | 0.002 | 0.798** | 0.842** | -0.044 |
| Indole-3-lactic acid | 0.000 | 0.001 | 0.001 | 0.998** | 1.187** | -0.189* |
| Gluconic acid | 0.006 | 0.004 | 0.004 | -0.454* | 2.830* | -0.052 |
| D-Mannitol | 0.031 | 0.006 | 0.003 | -2.408** | -3.239** | 0.831** |
| D-Maltose | 0.014 | 0.002 | 0.001 | -2.818** | -3.810** | 0.992** |
| D-Glucuronic acid | 0.001 | 0.002 | 0.002 | 0.331** | 0.392** | -0.061 |
| D-Fructose | 0.005 | 0.003 | 0.002 | -0.851** | -1.042** | 0.191** |
| Daidzein | 0.011 | 0.026 | 0.024 | 1.208** | 1.069** | 0.139 |
| Citric acid | 0.021 | 0.006 | 0.006 | -1.759** | -1.836** | 0.078 |
| 2-Hydroxycinnamic acid | 0.006 | 0.008 | 0.008 | 0.466** | 0.459** | 0.007 |

The major metabolites were selected based on at least one of fold-changes [log_2_ (T1/CT), log_2_ (T2/CT), log_2_ (T1/T2)] contrast was statistically significant. *0.001 < P < 0.05; **P < 0.001.
