# Supplementary material for: Comprehensive characterization of the bacterial community structure and metabolite composition of food waste fermentation products via microbiome and metabolome analyses
Source: PLoS One. 2022 Mar 15;17(3):e0264234. doi: 10.1371/journal.pone.0264234 (PMC9048815; doi:10.1371/journal.pone.0264234)
Supplement: S2 Table — (DOCX) [file pone.0264234.s003.docx]

**S2 Table. Identification of significant key metabolites by GC-MS in fermented food waste sample.**

|  | **Relative concenyration** | | | **Fold-changes** | | |
| --- | --- | --- | --- | --- | --- | --- |
| **Metabolite name** | **CT** | **T1** | **T2** | **Log_2_(T1/CT)** | **Log_2_(T2/CT)** | **Log_2_(T1/T2)** |
| Phenylethyl Alcohol | 0.007 | 0.029 | 0.043 | 1.939** | 2.509** | -0.571** |
| Acetic acid | 0.039 | 0.084 | 0.096 | 1.119** | 1.308** | -0.189 |
| 1-Butanol | 0.013 | 0.035 | 0.038 | 1.405** | 1.542** | -0.137** |
| β-Myrcene | 0.014 | 0.005 | 0.010 | -1.386** | -0.455** | -0.931** |
| Linalool | 0.035 | 0.015 | 0.024 | -1.196** | -0.560** | -0.637** |
| Geraniol | 0.007 | 0.001 | 0.001 | -3.287** | -3.390** | 0.102 |
| Eucalyptol | 0.014 | 0.003 | 0.004 | -2.005** | -1.649** | -0.357** |
| Anethole | 0.008 | 0.003 | 0.058 | -1.221** | 2.842** | -4.063** |
| 1-Hexanol | 0.005 | 0.002 | 0.003 | -1.363** | -0.745** | -0.619** |
| 2-Decenal | 0.005 | 0.008 | 0.004 | 0.659 | -0.428** | 1.088** |
| Oleic Acid | 0.010 | 0.016 | 0.006 | 0.704** | -0.691** | 1.395** |
| Hexanal | 0.002 | 0.005 | 0.002 | 1.555** | 0.336 | 1.219** |
| Propanoic acid | 0.004 | 0.043 | 0.026 | 3.454** | 2.719** | 0.735** |
| Octanoic acid | 0.005 | 0.013 | 0.019 | 1.332** | 1.850** | -0.518** |
| Phenol | 0.000 | 0.035 | 0.001 | 7.278** | 2.090** | 5.188** |
| Benzene | 0.002 | 0.000 | 0.001 | -3.421** | -0.983** | -2.438** |

The major metabolites were selected based on at least one of fold-changes [log_2_ (T1/CT), log_2_ (T2/CT), log_2_ (T1/T2)] contrast was statistically significant. *0.001 < P < 0.05; **P < 0.001.
