# Supplementary material for: Comprehensive characterization of the bacterial community structure and metabolite composition of food waste fermentation products via microbiome and metabolome analyses
Source: PLoS One. 2022 Mar 15;17(3):e0264234. doi: 10.1371/journal.pone.0264234 (PMC9048815; doi:10.1371/journal.pone.0264234)
Supplement: Attachment — Submitted filename: Response to Reviewers.docx [file pone.0264234.s004.docx]

Reviewer #1: This study intended to comprehensive characterize the bacterial community and metabolite composition of food waste fermentation products by microbiome and metabolome analyses. Overall the article presents enough data for a paper. The following suggestions should be helpful and considered for the significant improvement of the manuscript as well as their future study.

1. In the Abstract, the commercial bacterial agent and 2% of a laboratory-made bacterial agent can be simply introduced for more details.

**Answer:** Thank you for your suggestions. The required details have been included in the abstract as follows: “inoculated with or without 0.18% of a commercial bacterial agent consisting of multiple unknown strains and 2% of a laboratory-made bacterial agent consisting of *Enterococcus faecalis, Bacillus subtilis*, and *Candida utilis*.”

2. In the Abstract, the authors wrote “Microbial inoculation also affected the diversity and abundance of microbial communities”. However, how did the microbial inoculation affect the microbial abundance indices?

**Answer:** Thank you for your insightful observations. Microbial inoculation affected the community richness, and the Sobs and Ace indices were lower (P < 0.05) in the groups inoculated with the commercial bacterial agent and laboratory-made bacterial agent than in the non-inoculated treatment group. Microbial inoculation also affected the community diversity, and the Shannon index of the group inoculated with the laboratory-made bacterial agent was lower (P <0.05) than that of the group inoculated with the commercial bacterial agent and the uninoculated group.

3. The Introduction part should be improved. For example, the detailed amount of the production of the food waste in China can be mentioned.

**Answer:** Thank you for your suggestions. Liu et al. reported that food stalls, restaurants, and canteens in China generate approximately 45 million tons of food waste per year. This information has been included in the Introduction.

4. The authors should critically check the correctness of some descriptions. For example, the authors wrote that “There are no studies on the microbial communities and metabolic profiles of fermented food wastes of various sources and complex compositions processed in centralized treatment facilities” the microbial community should have been widely reported in my humble view since the Miseq sequencing has been widely used since 2011, while the food fermentation was also a common topic. So, it is too absolute to say that the microbial communities have not been reported.

**Answer:** Thank you for your suggestions. We agree that the presentation of that paragraph in the article was confusing. The point we were trying to make is that no one has yet analyzed the fermentation products of food waste treated in centralized treatment centers using 16SrRNA and metabolomic methods. We have improved the presentation of that section.

5. Why did the microbial inoculation reduce the alpha diversity indexes?

**Answer:** The commercial bacterial agent or laboratory-made bacterial agent added to the food waste medium are more likely to form the dominant flora. They will secrete some metabolites to prevent the growth and reproduction of others. Therefore, compared with the control group, the diversity of the inoculated treatment groups was reduced.

6. The reasons for the production of undesirable odors and some harmful substances can be explained based on the results.

**Answer:** Thank you for your question. Microbial inoculation reduced the pH value of the fermentation product, making the lipids with the benzene ring easier to hydrolyze, resulting in higher phenol production rates under acidic conditions. Additionally, *Acetobacter* was positively correlated with phenol and Unclassified was positively correlated with benzene. Hexanal, propionic acid, and octanoic acid (compounds with undesirable odors) may be produced mainly by the metabolism of some bacteria, such as *Acetobacter, Enterococcus*, and *Bacillus.*

7. The authors applied 0.18% of a commercial bacterial agent and 2% of a laboratory-made bacterial agent. Since 2% was ten times more than 0.18%, why did the authors choose a relatively high inoculation rate?

**Answer:** Thank you for your question. The commercial bacterial agent purchased is a freeze-dried product and the amount added for fermentation is a mass ratio. The addition of the commercial bacterial agent resulted in a larger increase in production costs. In order to save costs and facilitate production, the laboratory-made bacterial agent used in this experiment was collected by centrifugation and was not lyophilized.

8. The experimental setup seemed to be not described clearly, which can be presented in the SI.

**Answer:** Thank you for your suggestions. The relevant experimental setup has been described in detail in Figure S1.

Reviewer #2: Overview recommendation:
In this study, the microbial community and metabolite profile were investigated by 16S rRNA gene sequencing, GC-MS and LC-MS during the solid food waste fermented process. Meanwhile, the bacterial agent was used to improve the fermented products. The study is meaningful, however the description and analysis in the section of Results is not enough. And the cacography existed in the manuscript. Therefore, the paper need to be revised before publishing.

General recommendation:

1. Page 8, the section of Abstract: Suggest that the data related results should be added into the Abstract, rather than description without data.

**Answer:** Thank you for your suggestion. The related results have been included in the abstract.

2. Page 8, the section of Abstract: the name of genus, such as Leuconostoc, Lactococcus and Weissella, should be wrote in italic.

**Answer:** Thank you for your suggestion. We have changed these words to italics in the Abstract, as well as throughout our entire manuscript.

3. Page 9, the section of Introduction, the second paragraph: the sentence of “Chen collected food wasted…animal feeds was presented” is confuse, please revised it.

**Answer:** Thank you for your advice. I changed this sentence into “Three typical treatment processes (i.e., heat treatment, fermentation, and coupled hydrothermal treatment and fermentation) are usually used in centralized food waste treatment centers. Food waste processed using either of the aforementioned procedures is considered to have some nutritional value and meets relevant microbiological and chemical contaminant standards, making food waste a promising alternative to be used in animal diets.”

4. Page 11, the section of 2.1, the 5th line: the initial of “Samples” should be lower-case. The 8th line: “8% corn meal and 5% soybean meal powder” is mass ratio or volume ratio?

**Answer:** Thank you for your suggestions. The word "Samples" is now in lower case. Regarding the composition ratio of food waste and auxiliary materials, 87% was food waste heat treatment material, 8% was soybean meal, and 5% was cornmeal. These proportions are reported on a mass ratio basis.

5. The vertical spacing in Table 1 and Table 2 should be unification.

**Answer:** Thank you for your suggestions. The vertical spacing of Tables 1 and 2 is now equal.

6. The section of 3.6, the second paragraph, 6th-7th line: the initial of “1-hexanol” and “octanoic acid” should be the capital form. The 9th line: “Anethole” should not be italic, please revised it.

**Answer:** Thank you for your suggestions. All corrections have been implemented in the revised version of the manuscript.

7. The food waste samples was inoculated with 0.18% commercial inoculum 1 (T1) and 2% laboratory-made inoculum 2 (T2). Whether there is comparability? Because the additive amounts of bacterial agent were different.

**Answer:** Thank you for your question. The purchased commercial bacterial agent is available in lyophilized form and is added at a mass ratio of 0.18% of the food waste fermentation medium. The laboratory-made bacterial agent is obtained by centrifugal collection at 2% of the food waste fermentation medium. Many previous articles refer to the addition of centrifugal bacteria at 1–5% of the fermentation composition. In a preliminary study, we compared the fermentation effectiveness and cost of several different additions and decided to use a 2% inoculum.

8. How much is cost of additional bacterial agent when treatment of solid food waste fermented products? Is the price of bacterial agent acceptable?

**Answer:** Thank you for your questions. In the process of converting kitchen waste to feed, the cost of the bacterium is an important consideration. Adding 0.18% of bacteriological agent increases the treatment cost by an additional 120 RMB per ton. For solid waste treatment, the cost is too high and harder for companies to bear. In contrast, the laboratory-made bacterium formulation could be prepared by the factory itself. The liquid medium can be used for the homogenization of kitchen waste without resulting in additional pollution and waste. The increased cost is less than 1/5 of the commercial bacteriological agent.

9. Is the performance of bacterial agent durable?

**Answer:** We conducted colony counts of *Lactobacillus*, *Bacillus*, and yeasts in fermentation products obtained using the laboratory-made bacterial inoculant. The colony counts of *Lactobacillus* and *Bacillus* were within the same order of magnitude at 4, 14, and 21 days after fermentation. However, the number of yeast colonies after 14 and 21 days was significantly lower than that of colonies at 4 days post-fermentation.
